# Shining light on dinoflagellate photosystem I

**DOI:** 10.1038/s41467-024-47797-1

**Published:** 2024-04-18

**Authors:** Senjie Lin, Shuaishuai Wu, Jiamin He, Xiaoyu Wang, Arthur R. Grossman

**Affiliations:** 1 Department of Marine Sciences, University of Connecticut, Groton, CT 06340 USA; 2 College of Ocean and Earth Sciences, Xiamen University, Xiamen, Fujian 361102 China; 3 Department of Plant Biology, Carnegie Institution for Science, Palo Alta, CA 94305 USA; 4 Department of Biology, Stanford University, Palo Alta, CA 94305 USA

**Keywords:** Marine biology, Photosystem I, Structural biology

## Abstract

Dinoflagellates are ecologically important and essential to corals and other cnidarians as phytosymbionts, but their photosystems had been underexplored. Recently, photosystem I (PSI) of dinoflagellate *Symbiodinium* sp. was structurally characterized using cryo-Electron Microscopy (cryo-EM). These analyses revealed a distinct organization of the PSI supercomplex, including two previously unidentified subunits, PsaT and PsaU, and shed light on interactions between light harvesting antenna proteins and the PSI core. These results have implications with respect to the evolution of dinoflagellates and their association with cnidarians.

Dinoflagellates are a unique group of protists ubiquitously distributed in aquatic ecosystems. These algae are members of the Alveolata that represents a sister group to the parasitic Perkinsids and Apicomplexa. The dinoflagellates are a constant source of fascination because of their numerous remarkable and unusual characteristics^1,2^. Many dinoflagellates are photoautotrophic and significant contributors to global primary production, and species of the family Symbiodiniaceae are indispensable endosymbionts of stony corals, the foundation of coral reef ecosystems. Dinoflagellates are characterized by many non-canonical eukaryotic cytological features such as permanent condensation of chromosomes, abandonment of nucleosomes while maintaining the core histone genes (H2A, H2B, H3, and H4), and a closed mitosis and extranuclear spindle. Other remarkable features of dinoflagellates include the evolutionary history of their plastids and their associations with other organisms. For example, some dinoflagellates can derive energy from intracellular symbionts (e.g., *Kryptoperidinium foliaceum* with a diatom symbiont) or maintain a fully integrated plastid. The mature plastid can be either from a secondary endosymbiosis that gave rise to the typical chlorophyll *c*, peridinin lineage, from serial secondary endosymbioses that resulted in the chlorophyll *b*-containing lineage (e.g., *Lepidodinium chlorophorum*), or from tertiary replacement that led to the establishment of the haptophyte-type (fucoxanthin containing) plastid (e.g., *Karenia mikimotoi*). Additionally, some dinoflagellates possess plastids derived from secondary transient plastids (kleptoplasts) acquired from predation of either a cryptophyte^3^ and/or a haptophyte^4^. Those dinoflagellates with stable plastids have genomes that are typically comprised of heterogeneous plasmid-like minicircles that each contains a conserved noncoding region (presumably for replication and regulation) and 0, 1 or 2 genes that encode proteins with chloroplast associated functions^5,6^. Photosynthetic members of the family Symbiodiniaceae have evolved to support an endosymbiotic lifestyle, with activities reflecting both the functional and regulatory needs of the alga and their animal hosts. The structural organization, functional scope, and regulatory features of dinoflagellate plastids are still largely unexplored. A recent study^7^ using cryo-Electron Microscopy (cryo-EM) coupled with photochemical measurements has provided insights into the architecture of PSI, giving us a glimpse of the complex and distinct aspects of the dinoflagellate photosynthetic apparatus.

## The dinoflagellate PSI proteins, PsaT, and PsaU

**Fig. 1:**
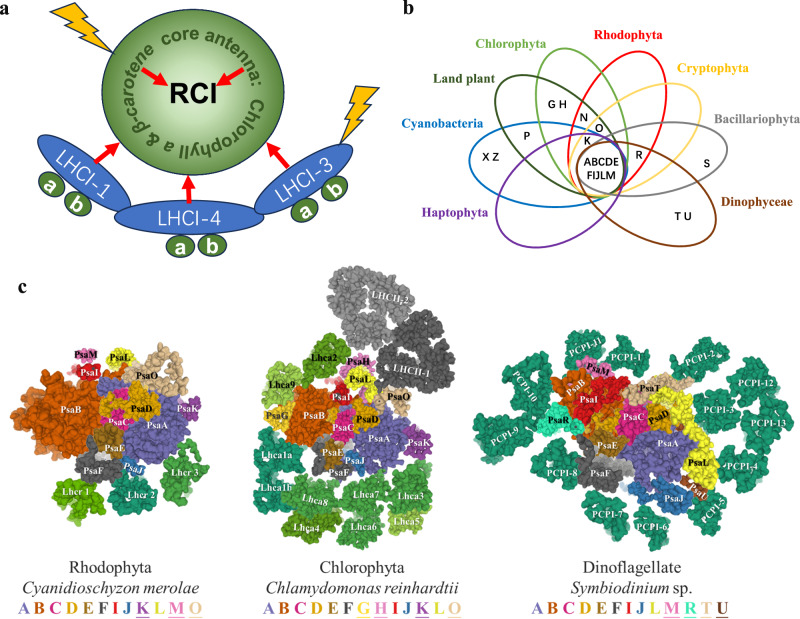
Photosystem I core proteins in plant model and algae. **a** Schematic plant model of structural and functional relationships of PSI reaction center (RCI) with core antenna (Chl *a* and β-carotene) and peripheral light-harvesting antenna (LHC), which are composed of pigment binding proteins bound to chl *a* and *b*. Only three LHC proteins are shown as examples. **b** Venn diagram showing ten common proteins and variable distinct proteins in each lineage. **c** Schematic of subunit distribution of PSI core and core antenna in Rhodophyta (PDB code: 6FOS), Chlorophyta (PDB code: 7D0J and 6JO5), and dinoflagellates (PDB code: 8JZF and 8JJR).

PSI absorbs light energy and fuels the light-induced transfer of electrons from reduced plastocyanin or cytochrome *c6* to oxidized ferredoxin. It consists of a core complex that binds chlorophyll (Chl) *a* and a peripheral antenna or light-harvesting complex I (LHCI) that binds both Chl *a* and Chl *b* (Viridiplantae) or Chl *c* (non-green lineages); excitation energy absorbed by the core and peripheral antenna is transferred to the reaction centers (Fig. 1a). In vascular plants, the PSI core is typically composed of 16 subunits, including Psa-A to -P^8^. PsaA and PsaB form a heterodimer that binds the primary electron donor P700 (chlorophyll *a* dimer) along with the electron acceptors A_0_ (Chl *a* molecule), A_1_ (phylloquinone), and F_X (_iron-sulfur cluster [4Fe-4S]). The remaining cofactors, F_A_ and F_B,_ are both iron-sulfur clusters associated with PsaC, which interacts with ferredoxin. The PSI core is highly conserved^9^, with ten common subunits shared between lineages of algae and vascular plants (Fig. 1b). In algae, the number of PSI core subunits ranges from 11 in haptophytes to 15 in green algae (only missing PsaP compared to land plants), although their structures are similar (Fig. 1c).

The report by ref. ^7^ sets dinoflagellates apart from other algae as they possess PSI complexes with the distinct polypeptides PsaT and PsaU; these proteins have not been reported in other photosynthetic organisms (Fig. 1). The dinoflagellate PSI core has 13 subunits; in addition to PsaT and PsaU, they contain ten subunits common to PSI in most other organisms and share PsaR with diatoms. Zhao et al. used cryo-EM to resolve the PSI core of a *Symbiodinium* sp. at 2.8 Å^7^. This analysis revealed regions of the density maps with high-resolution information that suggested distinct subunits potentially representing small peptides. The PSI polypeptide sequences deduced from the cryo-EM images were used for blast analyses to identify the genes encoding these proteins. Each of the candidate protein sequences identified was manually fitted to the high-resolution amino acid map of PSI, with the distinguishable amino acid side chains (phenylalanine, tyrosine, tryptophan, arginine, and glycine) used as anchors to identify the correct sequences from the pool of candidate sequences. This approach not only identified core PSI subunits (PsaA–F, PsaI, PsaJ, PsaL, PsaM, and PsaR) but also two previously unknown peptides, PsaT and PsaU. PsaT is on the stromal surface of the thylakoid membranes while PsaU is on its lumenal surface. The work clearly demonstrates how cryo-EM imaging can lead to de novo gene discoveries.

A similar cryo-EM study by ref. ^10^ also noted the presence of two unknown subunits in PSI of symbiodiniacean dinoflagellates, and the absence of these polypeptides in *Amphidinium carterae*, a non-symbiodiniacean species. Importantly, ref. ^7^ extended their observations by establishing the full sequences of these small polypeptides. Based on their structural analyses, the identified PsaT subunit interacts with various core subunits, including PsaB−D, PsaL, and PsaI, potentially aiding to stabilize the extrinsic subunits of the complex. Although PsaS in diatom PSI cores has a spatial position in common with PsaT, the structures of the two proteins differ significantly. Additionally, the introduction of PsaU into PSI leads to modifications of the loop structures of PsaA, facilitating their interactions with PsaM and three peridinin-Chl *a*/*c*-binding proteins, acpPCI-3 and acpPCI-5/6, thereby mediating the association of the core with acpPCI antenna proteins.

## Efficient electron transfer and energy quenching

In addition to the discovery of PsaT and PsaU, ref. ^7^ detected 13 peridinin-Chl *a*/*c*-binding proteins (acpPCIs) and noted distinct structural characteristics of the PSI–acpPCI supercomplex in *Symbiodinium* sp. compared to those of other organisms with plastids from the red algal lineage. These include elongated termini of subunits PsaD/E/I/J/L/M/R and acpPCI-1/3/5/7/8/11 that cause conformational alterations that are thought to enhance the association between the PSI core and peripheral antennae. For instance, the extended terminal loops of PsaL and PsaJ were predicted to interact with the antennae proteins acpPCI3 and acpPCI5/6, respectively (Supplementary Fig. 10 in ref. ^7^). The elongated termini of PsaI/J/L/M/R, combined with the PsaT subunit, were predicted to establish a protective layer enveloping the stromal side of the PSI core. This configuration minimizes interactions between the reaction center and stromal molecules, potentially reducing damage from reactive oxygen species and safeguarding reaction center pigments during high-light exposure. Additionally, these elongated termini may allow for the formation of a distinct PsaC/D/E-ferredoxin domain, enhancing the binding affinity of ferredoxin for PSI. The extended N-terminal domains of PsaL and PsaJ, positioned along the stromal surface of PsaA, interact with each other and create a molecular thread that binds the stromal loops of acpPCI-3, acpPCI-5, and acpPCI-6, enhancing the cohesion of the acpPCI antennae. The PsaU subunit spans the edges of PsaA and PsaB on their lumenal surfaces, leaving the binding sites for cytochrome c6 unobstructed. Similarly, the extended N- or C-termini of other core proteins presumably promote interactions with neighboring subunits. In addition, distinct chlorophyll and carotenoid molecules were found in various positions of the PSI-acpPCI complex, which were predicted to function in the dissipation of excessive absorbed light energy. These structural characteristics likely facilitate effective interactions and intermolecular electron transfer between PSI subunits and other electron carriers, thereby conferring dinoflagellates with the ability to respond rapidly to light fluctuations and to withstand high light exposure when close to the ocean surface, as occurs in their associations with tropical corals.

## Questions for future research

The cryo-EM study creates avenues for further inquiry into critical aspects of dinoflagellate photosynthesis. First, it is important to determine whether the PSI core of dinoflagellates is complete with the inclusion of the PsaT and PsaU subunits. The composition and organization of PSI subunits may change in response to light conditions, potentially explaining why PsaO, although present in plants, was not detected by cryo-EM. Could the apparent absence of PsaO in plants represent a specific photochemical state of PSI? More work is needed to determine how the architecture of PSI described by ref. ^7^ and ref. ^10^ might vary under different light and nutrient (including inorganic carbon) conditions. The combination of genomics, molecular biology, and time-serial cryo-EM modeling will prove powerful in reconstructing a complete repertoire of PSI core proteins, their interactions and the potential functional consequences of these interactions.

Second, PsaT and PsaU represent recently discovered components of PSI, although we know almost nothing about their occurrence in dinoflagellate species outside of the Symbiodiniaceae, or in other groups of algae. ref. ^10^ found that they were absent in some non-symbiodiniacean species such as *A. carterae*, raising the possibility that PsaT and PsaU are distinct to coral phytosymbionts. A systematic survey into dinoflagellate genomes and transcriptomes is needed to determine whether PsaT and PsaU are present in other dinoflagellates and how they have evolved. The sequences identified in ref. ^7^ will be instrumental for such efforts.

Third, based on current literature, PsaO does not seem to be present in dinoflagellates^7^. PsaO was only discovered about two decades ago as a small (10-kDa) protein with two transmembrane helices in the plant model *Arabidopsis thaliana*^11^. Using RNA interference, it was shown that PsaO was involved in state transitions, which involves the partitioning of light-harvesting complex II (LHCII) antennae subunits between PSI and PSII. This process helps balance energy capture by the two photosystems and enhances photosynthetic efficiency. PsaO is positioned close to PsaL on the PsaH/L/I side of PSI, with PsaH, PsaL, PsaO, and possibly PsaI all contributing to the formation of a domain of PSI involved in interacting with light-harvesting complex II (LHCII)^12^. PsaO has been found in various plants, green algae, rhodophytes, and cryptophytes but, so far, not in diatoms^7,13–15^. The cryo-EM analysis by ref. ^7^ was also unable to identify PsaO in *Symbiodinium* sp. However, as alluded to earlier, cryo-EM analysis did not detect this protein in plants, even though other methods did^8^. Interestingly, our blast analysis using the *A. thaliana* PsaO sequence as the query revealed homologs (from 7e–19 to 7e–25, with sequence coverage of 62.9–80.7%) in two dinoflagellate species (*Alexandrium tamarense*, *Cladocopium goreaui*). Experimental verification for these results and a more systematic investigation of the prevalence of PsaO in dinoflagellates is needed.

Fourth, given the complex evolutionary history of dinoflagellate plastids, it is of interest to determine whether non-peridinin plastids derived from serial secondary endosymbiosis and tertiary replacement have undergone convergent evolution in which their original (chlorophyte or haptophyte) plastids evolved to attain features similar to those of peridinin-containing plastids. A recent study has shown that sequences of non-peridinin dinoflagellate plastid proteins have evolved more rapidly than counterparts in their source lineages (chlorophytes and haptophytes), becoming more comparable to peridinin plastid proteins^16^. These findings suggest a host effect on plastid evolution. Cryo-EM analyses of organisms with non-peridinin plastids will likely provide further insights into the structure, function, and evolution of PSI in the diverse dinoflagellate lineages.

Finally, how the distinct PSI architecture and protein composition confer (presumably) a competitive advantage to dinoflagellates in the environment warrants further research. During the summer months, when illumination can be intense, dinoflagellates often outgrow other algae in the ocean. On the one hand, polar species such as *P. glacialis* must endure extended periods of largely complete darkness in the winter. Additionally, corals harboring symbiodiniacean endosymbionts live in the tropical surface oceans where photoprotection is crucial for maintaining ecosystem health. The findings of ref. ^7^ will help us better understand the structure and function of PSI in dinoflagellates and potentially, how the photosynthetic apparatus has evolved to support the endosymbiotic lifestyle of the Symbiodiniaceae and coral reef ecology.
